# Supplementary material for: Multi-Omics Analysis Reveals Age-Related Microbial and Metabolite Alterations in Non-Human Primates
Source: Microorganisms. 2023 Sep 26;11(10):2406. doi: 10.3390/microorganisms11102406 (PMC10609416; doi:10.3390/microorganisms11102406)
Supplement: Supplementary file 1 [file microorganisms-11-02406-s001.zip › microorganisms-2621587-supplementary.pdf]

Supplementary files

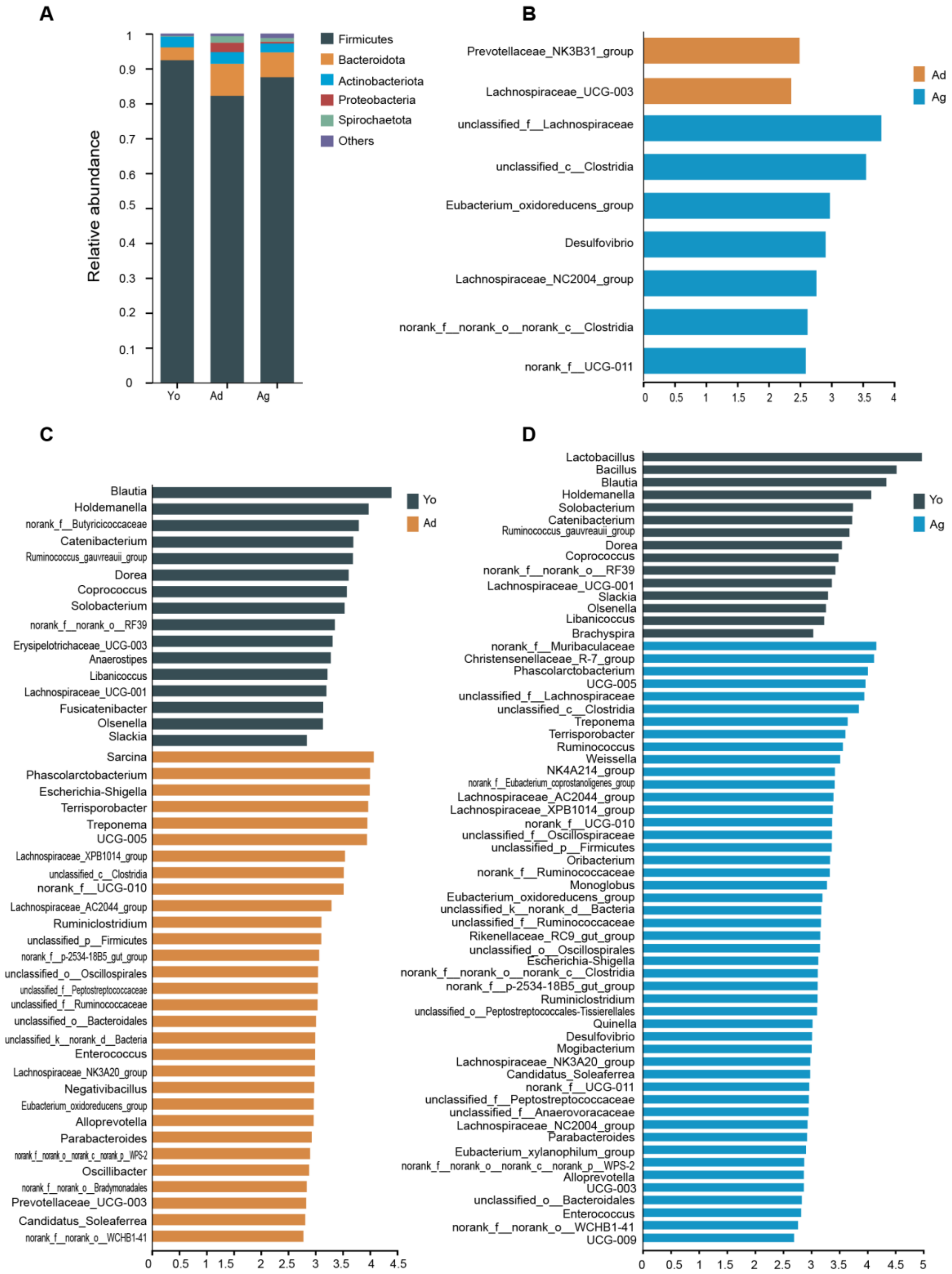

Figure S1. The composition of gut microbiota and discriminative genera observed in pairwise comparisons among juvenile, adult and old groups. (**A**) Bar plots showing the relative abundance of microbiota of three groups at phylum level. (**B**) 9 genera attributed to distinguishing adult from oldness. (**C**) 46 genera were responsible for distinguishing juvenile from adult. (**D**) 63 genera ac-counted for distinguishing juvenile from oldness. Differentiating

gut genera identified by LefSe with LDA effect size ≥2 and $P \leq 0.05$. Yo, juvenile, n = 9; Ad, adult, n = 12; Ag, oldness, n = 15.

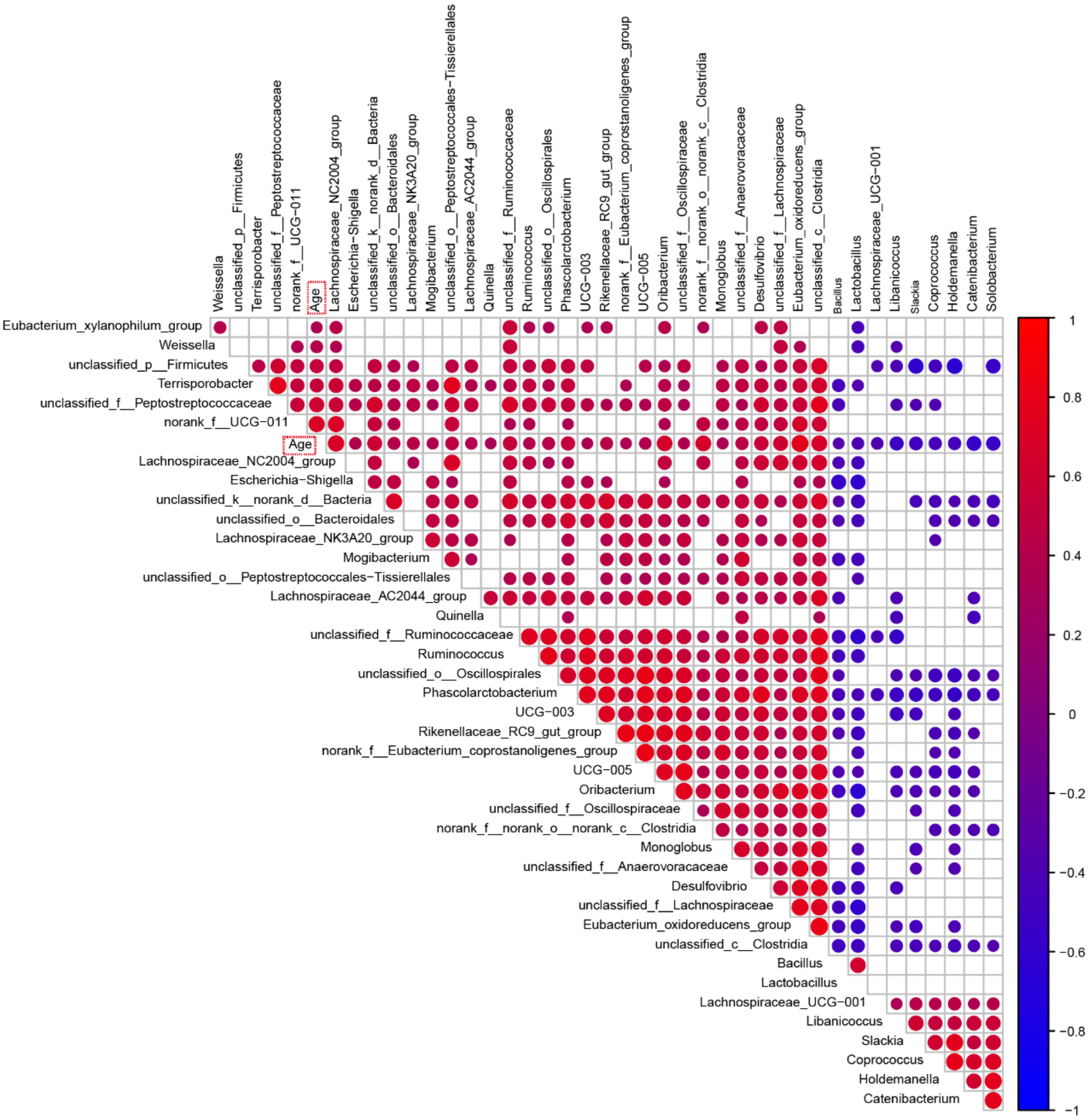

Figure S2. Correlation analysis of differential gut microbiota with age. The microbes used for cor-relation calculations met the criterion of significant differences between at least two age groups (see Figures S1B-D). In total, there are 41 discriminative microbes significantly correlated with age (the heatmap exclusively displayed age-related microbes). The size and color of each scatter plot point showed the *p* values of Spearman's correlation and correlation coefficient values (red points rep-resented positive correlations, $P < 0.05$ and $R > 0.3$; blue points represented negative correlations, $P < 0.05$ and $R < -0.3$).

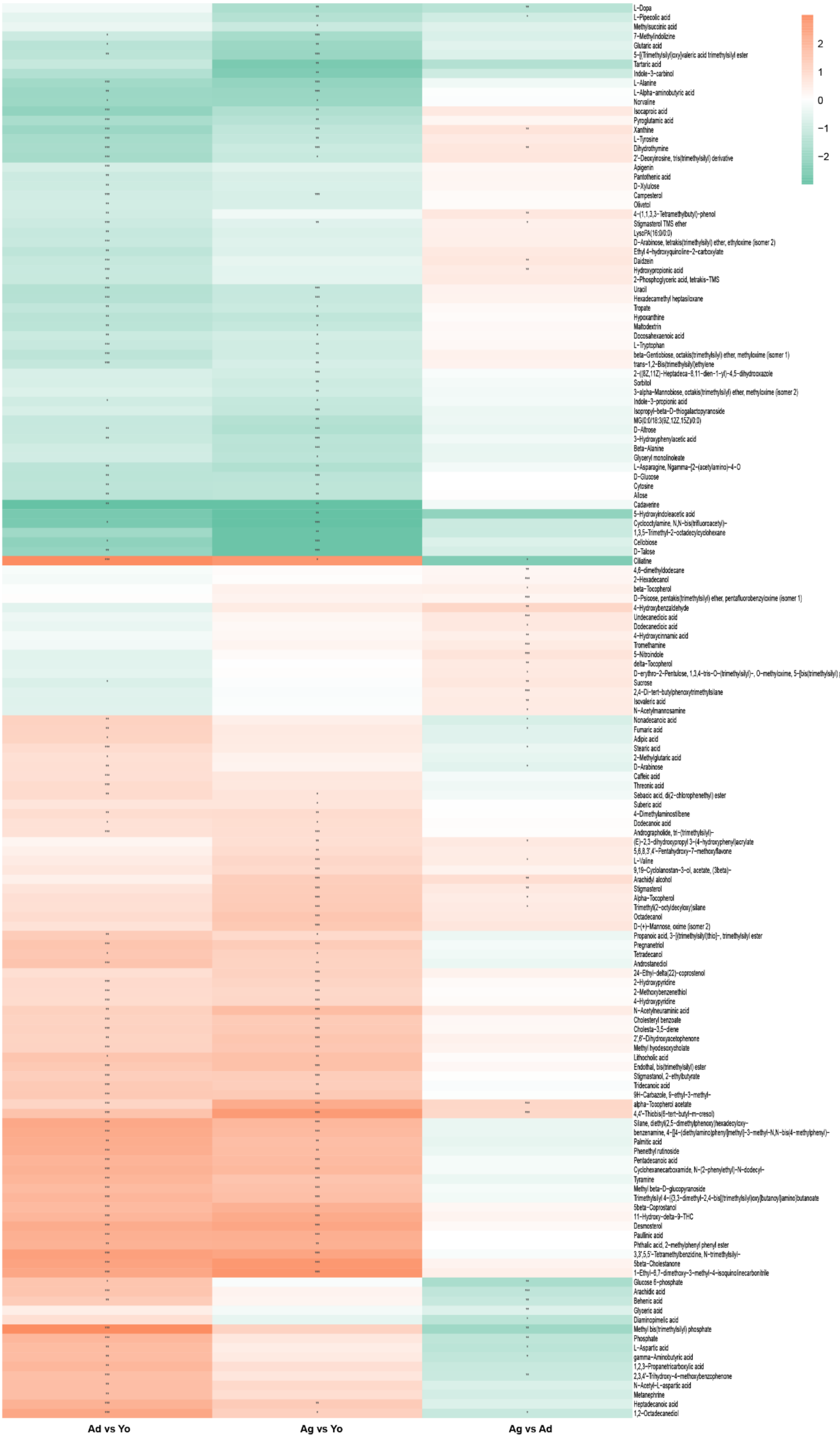

L-Dopa
L-Pipecolic acid
Methylsuccinic acid
7-Methylindolizine
Glutaric acid
5-[(Trimethylsilyl)oxy]valeric acid trimethylsilyl ester
Tartaric acid
Indole-3-carbinol
L-Alanine
L-Alpha-aminobutyric acid
Norvaline
Isocaproic acid
Pyroglutamic acid
Xanthine
L-Tyrosine
Dihydrothymine
2'-Deoxyinosine, tris(trimethylsilyl) derivative
Apigenin
Pantothenic acid
D-Xylulose
Campesterol
Olivetol
4-(1,1,3,3-Tetramethylbutyl)-phenol
Stigmasterol TMS ether
LysoPA(16:0/0:0)
D-Arabinose, tetrakis(trimethylsilyl) ether, ethyloxime (isomer 2)
Ethyl 4-hydroxyquinoline-2-carboxylate
Daidzein
Hydroxypropionic acid
2-Phosphoglyceric acid, tetrakis-TMS
Uracil
Hexadecamethyl heptasiloxane
Tropate
Hypoxanthine
Maltodextrin
Docosahexaenoic acid
L-Tryptophan
beta-Gentiobiose, octakis(trimethylsilyl) ether, methyloxime (isomer 1)
trans-1,2-Bis(trimethylsilyl)ethylene
2-((8Z,11Z)-Heptadeca-8,11-dien-1-yl)-4,5-dihydrooxazole
Sorbitol
3-alpha-Mannobiose, octakis(trimethylsilyl) ether, methyloxime (isomer 2)
Indole-3-propionic acid
Isopropyl-beta-D-thiogalactopyranoside
MG(0:0/18:3(9Z,12Z,15Z)/0:0)
D-Altrose
3-Hydroxyphenylacetic acid
Beta-Alanine
Glyceryl monolinoleate
L-Asparagine, Ngamma-[2-(acetylamino)-4-O
D-Glucose
Cytosine
Allose
Cadaverine
5-Hydroxyindoleacetic acid
Cyclooctylamine, N,N-bis(trifluoroacetyl)-
1,3,5-Trimethyl-2-octadecylcyclohexane
Cellobiose
D-Talose
Ciliatine
4,6-dimethyldodecane
2-Hexadecanol
beta-Tocopherol
D-Psicose, pentakis(trimethylsilyl) ether, pentafluorobenzyloxime (isomer 1)
4-Hydroxybenzaldehyde
Undecanedicic acid
Dodecanedioic acid
4-Hydroxycinnamic acid
Tromethamine
5-Nitroindole
delta-Tocopherol
D-erythro-2-Pentulose, 1,3,4-tris-O-(trimethylsilyl)-, O-methyloxime, 5-[bis(trimethylsilyl) phosphate]
Sucrose
2,4-Di-tert-butylphenoxytrimethylsilane
Isovaleric acid
N-Acetylmannosamine
Nonadecanoic acid
Fumaric acid
Adipic acid
Stearic acid
2-Methylglutaric acid
D-Arabinose
Caffeic acid
Threonic acid
Sebacic acid, di(2-chlorophenethyl) ester
Suberic acid
4-Dimethylaminostilbene
Dodecanoic acid
Andrographolide, tri-(trimethylsilyl)-
(E)-2,3-dihydroxypropyl 3-(4-hydroxyphenyl)acrylate
5,6,8,3',4'-Pentahydroxy-7-methoxyflavone
L-Valine
9,19-Cyclolanostan-3-ol, acetate, (3beta)-
Arachidyl alcohol
Stigmasterol
Alpha-Tocopherol
Trimethyl(2-octyldecyloxy)silane
Octadecanol
D-(+)-Mannose, oxime (isomer 2)
Propanoic acid, 3-[(trimethylsilyl)thio]-, trimethylsilyl ester
Pregnanetriol
Tetradecanol
Androstanediol
24-Ethyl-delta(22)-coprostenol
2-Hydroxypyridine
2-Methoxybenzenethiol
4-Hydroxypyridine
N-Acetylneuraminic acid
Cholesteryl benzoate
Cholesta-3,5-diene
2',6'-Dihydroxyacetophenone
Methyl hyodesoxycholate
Lithocholic acid
Endothal, bis(trimethylsilyl) ester
Stigmastanol, 2-ethylbutyrate
Tridecanoic acid
9H-Carbazole, 9-ethyl-3-methyl-
alpha-Tocopherol acetate
4,4'-Thiobis(6-tert-butyl-m-cresol)
Silane, diethyl(2,5-dimethylphenoxy)hexadecyloxy-
benzenamine, 4-[[4-(diethylamino)phenyl]methyl]-3-methyl-N,N-bis(4-methylphenyl)-
Palmitic acid
Phenethyl rutinoside
Pentadecanoic acid
Cyclohexanecarboxamide, N-(2-phenylethyl)-N-dodecyl-
Tyramine
Methyl beta-D-glucopyranoside
Trimethylsilyl 4-({3,3-dimethyl-2,4-bis[(trimethylsilyl)oxy]butanoyl}amino)butanoate
5beta-Coprostanol
11-Hydroxy-delta-9-THC
Desmosterol
Paullinic acid
Phthalic acid, 2-methylphenyl phenyl ester
3,3',5,5'-Tetramethylbenzidine, N-trimethylsilyl-
5beta-Cholestanone
1-Ethyl-6,7-dimethoxy-3-methyl-4-isoquinolinecarbonitrile
Glucose 6-phosphate
Arachidic acid
Behenic acid
Glyceric acid
Diaminopimelic acid
Methyl bis(trimethylsilyl) phosphate
Phosphate
L-Aspartic acid
gamma-Aminobutyric acid
1,2,3-Propanetricarboxylic acid
2,3,4'-Trihydroxy-4-methoxybenzophenone
N-Acetyl-L-aspartic acid
Metanephrine
Heptadecanoic acid
1,2-Octadecanediol
2
1
0
−1
−2
Ad vs Yo
Ag vs Yo
Ag vs Ad

Figure S3. Changes in fecal metabolites discerned from pairwise comparisons among the juvenile, adult, and old groups. A total of 151 fecal metabolites showed significant differences between at least two age groups. The statistical significance was denoted on the squares (**p* < 0.05 and VIP > 1; ***p* < 0.01 and VIP > 1; ****p* < 0.001 and VIP > 1). In pairwise comparisons, red squares indicate metabolites were significantly up-regulated in the higher-age group (logarithmic transformation of fold change values > 0), while green squares indicate those were significantly down- regulated (logarithmic transformation of fold change values < 0) compared to the lower-age group. Yo, juvenile, n = 7; Ad, adult, n = 10; Ag, oldness, n = 13.

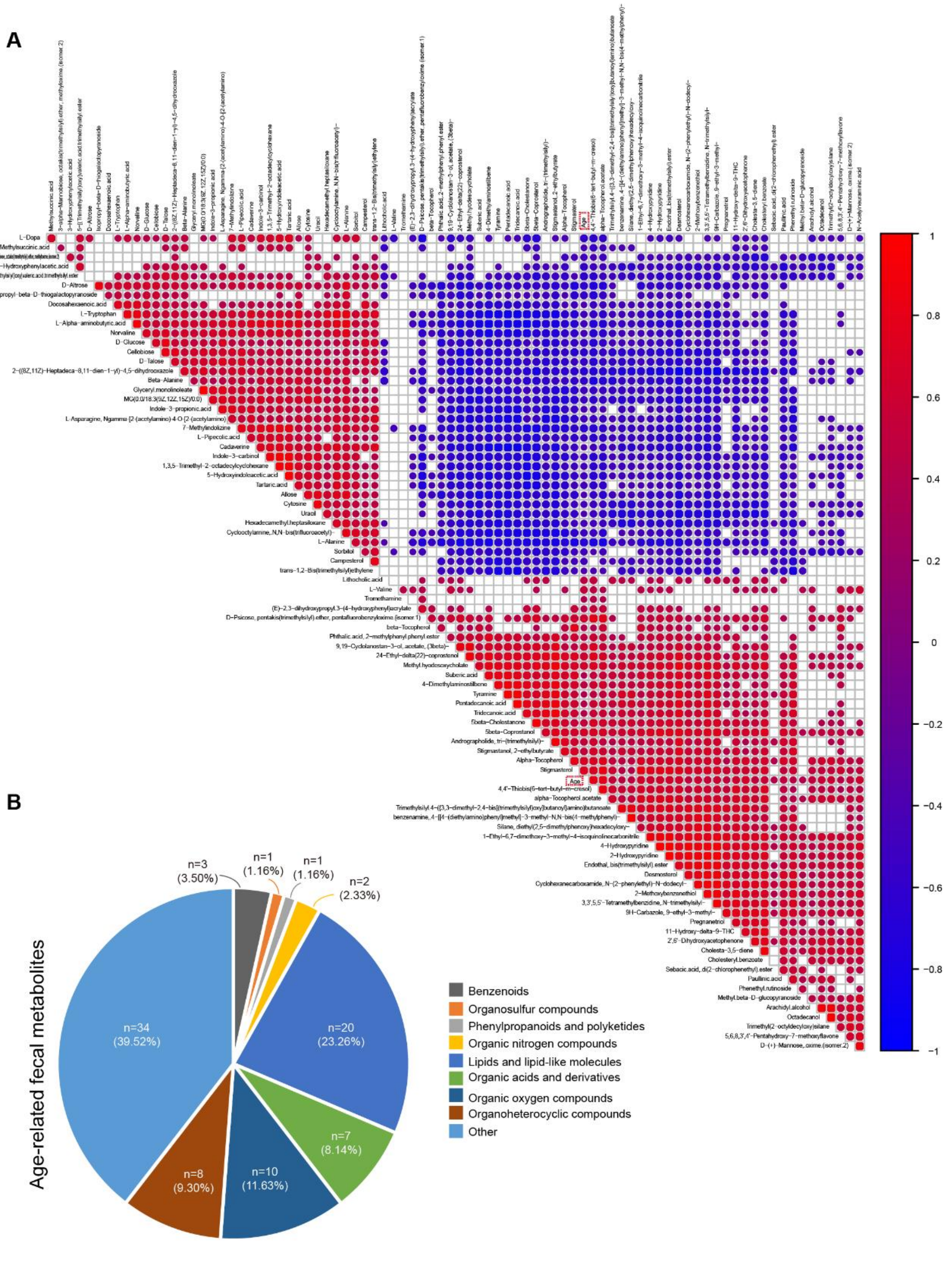

Figure S4. Correlation analysis of differential fecal metabolites with age. The metabolites used for correlation calculations met the criterion of significant differences between at least two age groups (see Figures S2). (**A**) There are a total of 86 differential metabolites significantly correlated with age (the heatmap exclusively displayed age-related metabolites). The size and color of each scatter plot point showed the *p* values of Spearman's correlation and correlation coefficient values (red points represented positive correlations, $P < 0.05$ and $R > 0.3$; blue points represented negative correlations, $P < 0.05$ and $R < -0.3$). (**B**) A pie chart illustrated the 86 age-related metabolites mainly belong to four categories: lipids and lipid-like molecules, organic oxygen compounds, organic acids and derivatives, and organoheterocyclic compounds.

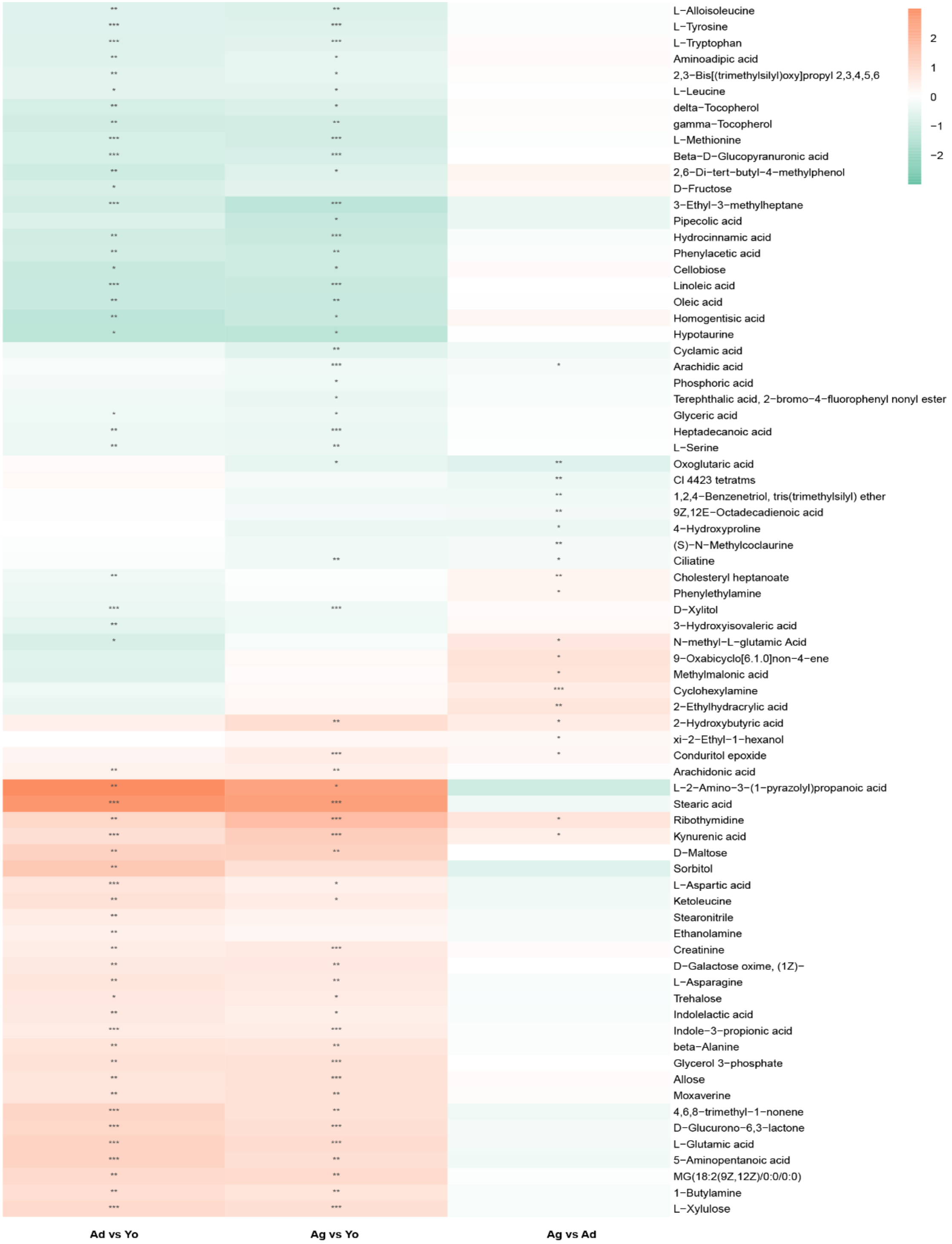

Figure S5. Alterations in serum metabolites observed in pairwise comparisons among juveniles, adults and old groups. A total of 75 fecal metabolites showed significant differences between at least two age groups. The statistical significance was denoted on the squares (* $p < 0.05$ and VIP > 1; ** $p < 0.01$ and VIP > 1; *** $p < 0.001$ and VIP > 1). In pairwise comparisons, red squares indicate metabolites were significantly up-regulated in the higher-age group (logarithmic transformation of fold change values > 0), while green squares indicate those were significantly down- regulated (logarithmic transformation of fold change values < 0) compared to the lower-age group. Yo, juvenile, n = 9; Ad, adult, n = 12; Ag, oldness, n = 15.

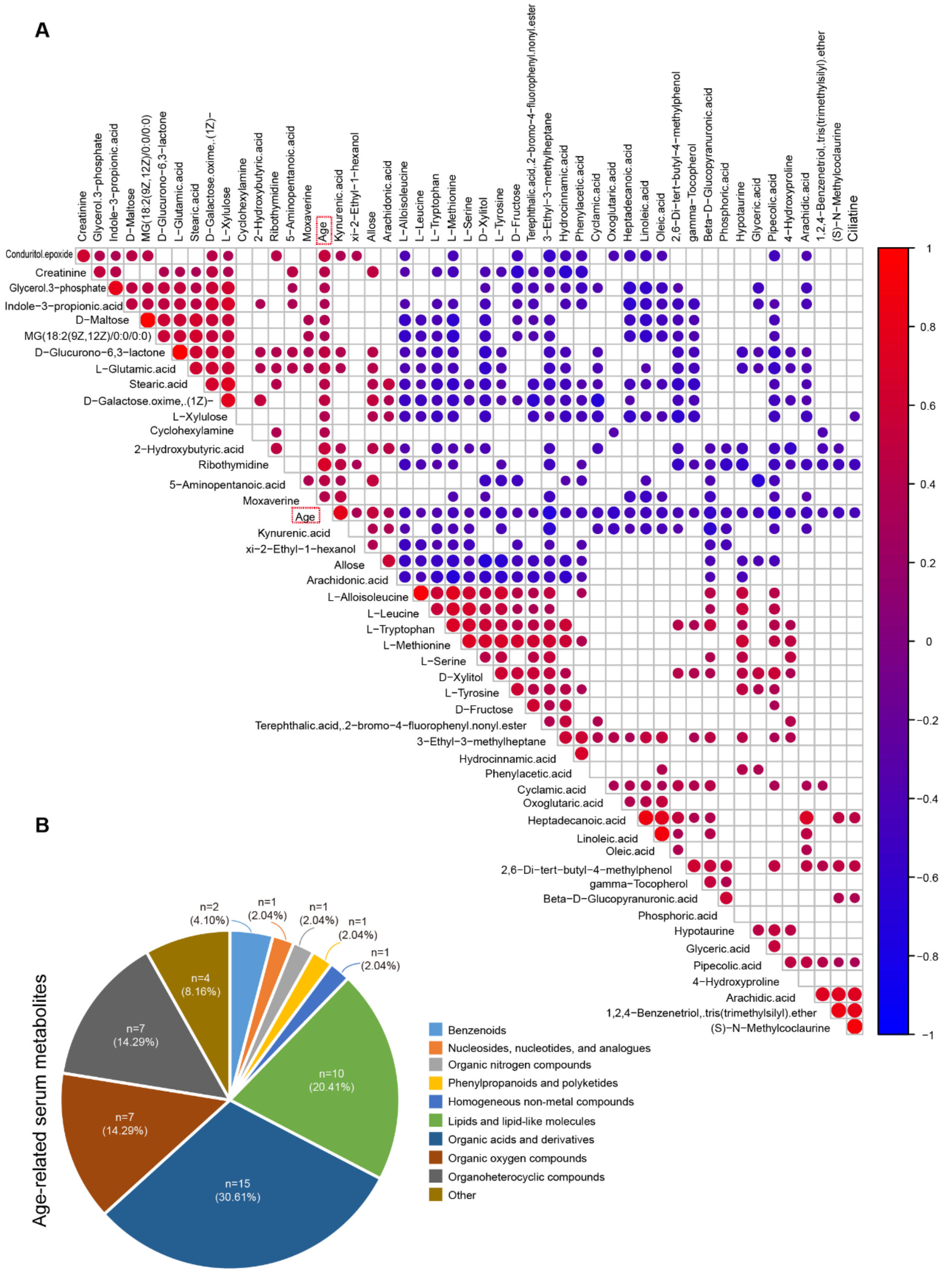

Figure S6. Correlation analysis of differential serum metabolites with age. The metabolites used for correlation calculations met the criterion of significant differences between at least two age groups (see Figures S3). (A) There are a total of 49 differential metabolites significantly correlated with age (the heatmap exclusively displayed age-related metabolites). The size and color of each scatter plot point showed the *p* values of Spearman's correlation and correlation coefficient values (red points represented positive correlations, $P < 0.05$ and $R > 0.3$; blue points represented negative correlations, $P < 0.05$ and $R < -0.3$). (B) Consistent with the fecal

metabolome, a pie chart revealed the 49 age-related metab-olites mainly belong to four categories, which are organic acids and derivatives, lipids and lipid-like molecules, organic oxygen compounds, and organoheterocyclic compounds.

Table S1. The detail characteristics of the recruited rhesus macaques.

Sample ID	Gender	Age	Stage	Dugs-Free #	Health Status #	Host	Geographical Region *
Yo1	Male	3	Juvenile	Yes	Normal	Chinese	Xishan
Yo2	Male	3	Juvenile	Yes	Normal	Chinese	Xishan
Yo3	Male	3	Juvenile	Yes	Normal	Chinese	Xishan
Yo4	Male	3	Juvenile	Yes	Normal	Chinese	Xishan
Yo5	Male	3	Juvenile	Yes	Normal	Chinese	Xishan
Yo6	Male	3	Juvenile	Yes	Normal	Chinese	Xishan
Yo7	Male	3	Juvenile	Yes	Normal	Chinese	Xishan
Yo8	Male	3	Juvenile	Yes	Normal	Chinese	Xishan
Yo9	Male	3	Juvenile	Yes	Normal	Chinese	Xishan
Ad1	Male	9	Adult	Yes	Normal	Chinese	Xishan
Ad2	Male	9	Adult	Yes	Normal	Chinese	Xishan
Ad3	Male	11	Adult	Yes	Normal	Chinese	Xishan
Ad4	Male	11	Adult	Yes	Normal	Chinese	Xishan
Ad5	Male	9	Adult	Yes	Normal	Chinese	Xishan
Ad6	Male	12	Adult	Yes	Normal	Chinese	Xishan
Ad7	Male	13	Adult	Yes	Normal	Chinese	Xishan
Ad8	Male	8	Adult	Yes	Normal	Chinese	Xishan
Ad9	Male	10	Adult	Yes	Normal	Chinese	Xishan
Ad10	Male	10	Adult	Yes	Normal	Chinese	Xishan
Ad11	Male	8	Adult	Yes	Normal	Chinese	Xishan
Ad12	Male	15	Adult	Yes	Normal	Chinese	Xishan
Ag1	Male	26	Elderly	Yes	Normal	Chinese	Xishan
Ag2	Male	20	Elderly	Yes	Normal	Chinese	Xishan
Ag3	Male	23	Elderly	Yes	Normal	Chinese	Xishan
Ag4	Male	26	Elderly	Yes	Normal	Chinese	Xishan
Ag5	Male	16	Elderly	Yes	Normal	Chinese	Xishan
Ag6	Male	24	Elderly	Yes	Normal	Chinese	Xishan
Ag7	Male	23	Elderly	Yes	Normal	Chinese	Xishan
Ag8	Male	25	Elderly	Yes	Normal	Chinese	Xishan
Ag9	Male	23	Elderly	Yes	Normal	Chinese	Xishan
Ag10	Male	22	Elderly	Yes	Normal	Chinese	Xishan
Ag11	Male	24	Elderly	Yes	Normal	Chinese	Xishan
Ag12	Male	23	Elderly	Yes	Normal	Chinese	Xishan
Ag13	Male	22	Elderly	Yes	Normal	Chinese	Xishan
Ag14	Male	18	Elderly	Yes	Normal	Chinese	Xishan
Ag15	Male	16	Elderly	Yes	Normal	Chinese	Xishan

Confirmation based on veterinary diagnosis and quarantine records. * The region is located in the temperate zone and has a subtropical monsoon marine climate (E 120º19′ 19″N 31º 07′05″).
